# Anlotinib for the Treatment of Multiple Recurrent Lumbar and Sacral Cord Hemangioblastomas: A Case Report

**DOI:** 10.3389/fonc.2022.859157

**Published:** 2022-04-27

**Authors:** Nan Jin, Chunxiao Sun, Yijia Hua, Xinyu Wu, Wei Li, Yongmei Yin

**Affiliations:** ^1^ Department of Oncology, The First Affiliated Hospital of Nanjing Medical University, Nanjing, China; ^2^ The First Clinical College of Nanjing Medical University, Nanjing, China; ^3^ Jiangsu Key Lab of Cancer Biomarkers, Prevention and Treatment, Collaborative Innovation Center for Personalized Cancer Medicine, Nanjing Medical University, Nanjing, China

**Keywords:** anlotinib, hemangioblastoma, anti-angiogenesis, case report, tyrosine kinase inhibitor (TKI)

## Abstract

**Background:**

Hemangioblastoma (HB) is a rare and highly vascularized tumor that originates from the central nervous system as well as other part of the body. They can appear sporadically or as part of von Hippel–Lindau (VHL) disease, a rare hereditary cancer syndrome. Although surgery can cure the majority of HBs, the disease shows a treatment-refractory challenge upon recurrence. HBs express a high amount of vascular endothelial growth factor (VEGF) which is responsible for angiogenesis and subsequently tumor progression. Anti-angiogenic treatment like bevacizumab has showed effect on HB, so we hypothesized that anlotinib could trigger HB regression *via* its inhibitory effect on VEGF.

**Case Presentation:**

We will share our experience in treating a 62-year-old woman with multiple recurrent lumbar and sacral cord HBs. She was treated with anlotinib (8mg qd d1-14, q3w) for three months and her follow up radiological examination demonstrated marked tumor regression which was evaluated as having partial response pursuant to RECIST 1.1 system. She is currently still receiving treatment of anlotinib orally and the lesions continuously reduced.

**Conclusion:**

We have reported that anlotinib can cause significant radiographic response in a patient with multiple recurrent lumbar and sacral cord HBs for the first time. This might enable a novel therapeutic approach for patients with multiple recurrent HB or those with multiple lesions such as in VHL disease which are difficult to resect surgically.

## Introduction

Hemangioblastoma (HB) is a rare and highly vascularized tumor that originates from the central nervous system (CNS) as well as other part of the body. HBs located in the CNS commonly arise in the brain and less commonly in the spinal cord. Most of HB lesions are sporadic and approximately account for 75% of cases, whereas in 25% of patients, they are manifestations of von Hippel-Lindau (VHL) disease and are often multifocal (1). HBs can result in significant neurological dysfunction by mass effect or hemorrhage in spite of their slow-growing benign nature. These tumors express a high amount of vascular endothelial growth factor (VEGF) which drives angiogenesis, a process that explains their highly vascular nature (2). The traditional treatment for HBs within accessible locations is surgical resection (3), leading to a cure typically. Nonetheless, up to 25% of HB patients have recurrence after surgical extirpation on the basis of past statistics (4, 5). For patients with recurrent HB, repeated surgical procedures are less recommended and surgery should be reserved as a salvage modality. Radiation therapy (RT) is performed following surgery for incompletely resected or recurrent lesions. However, it increases risks of radiation necrosis owing to overlapped irradiation zones (6). To date, targeted treatments and chemotherapy play a limited role in recurrent HB (7).

Given that HBs express high levels of VEGF, it appeals to be a promising target to block angiogenesis for controlling tumor proliferation conceptually. Anti-angiogenic agents like bevacizumab have showed significant clinical benefit on a 51-year-old man with a surgically unresectable cervical cord HB (8). Anlotinib, a novel orally administered tyrosine kinase inhibitor (TKI), is characterized as a potent and highly selectively vascular endothelial growth factor receptor (VEGFR)-2 inhibitor (9). Therefore, recurrent HB patients could derive benefit from anlotinib logically. Anlotinib has been reported to inhibit pathological ocular neovascularization (10), improve the life quality of patients with advanced non-small cell lung cancer (NSCLC) (11) and so on. Furthermore, previous literatures have demonstrated that anlotinib possesses additional advantages, including well bioavailability and tolerable safety profiles (12). But as far as we know, there are no published reports regarding the administration of this agent in HB. In this report, we will show tumor regression and meaningful clinical improvement responding to anlotinib in a patient with multiple recurrent lumbar and sacral cord HB.

## Case Presentation

**Figure 1:**
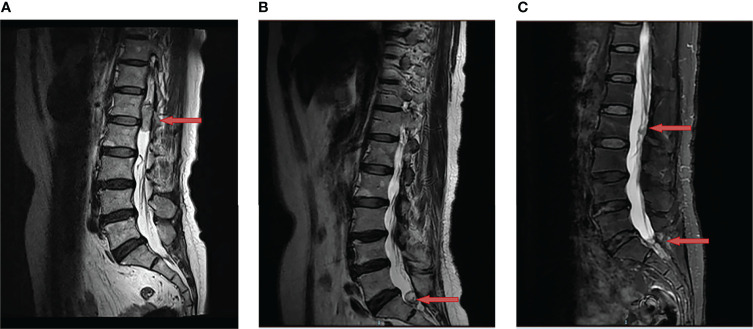
Changes of hemangioblastoma of the magnetic resonance imaging (MRI). **(A)** A space-occupying lesion at L1-L2 was detected (red arrow) in May 2016; **(B)** A recurrent site at S1 was observed (red arrow) after the first surgery in July 2019; **(C)** Two recurrent sites at L2 and S1-S2 were discovered (red arrow) after the second surgery in June 2020.

A 62-year-old woman came to our department for a consultation due to multiple recurrent HBs. In May 2016, she presented to our neurosurgery with a half-year history of persistent lower back pain that was radiating to left leg. She was initially diagnosed with lumbar disk herniation and received symptomatic treatment, but the clinical condition continued to deteriorate. The magnetic resonance imaging (MRI) showed a space-occupying lesion at L1-L2 (**Figure 1A**). Then she underwent a complete surgical resection under general anesthesia and symptoms were significantly alleviated after the operation. Histology confirmed the diagnosis of HB and immunohistochemical results showed EMA (-), S-100 (-), GFAP (-), Vimentin (++) and Ki-67 (10-15%). The patient has no similar family history and no other lesions characteristic of VHL disease were found, so she was diagnosed as a sporadic HB case. A MRI was done every six months postoperatively to follow up.

In July 2019, the patient was admitted to our clinic again due to a one-year history of left leg pain which progressively worsened over the last three months with difficulty in urination and defecation. She underwent a MRI of the lumbar spine which showed a space-occupying lesion at S1 (**Figure 1B**), thus the tumor resection was performed again. Immunohistochemical results showed EMA (-), S-100 (-), GFAP (-) and Ki-67 (sporadic +). Recurrent HB was diagnosed according to pathological results and medical history. Postoperatively, her pain significantly relieved. In June 2020, MRI indicated tumor recurrence at L2 and S1-S2 (**Figure 1C**). The patient did not receive any treatment due to free of symptoms in the next one year.

**Figure 2:**
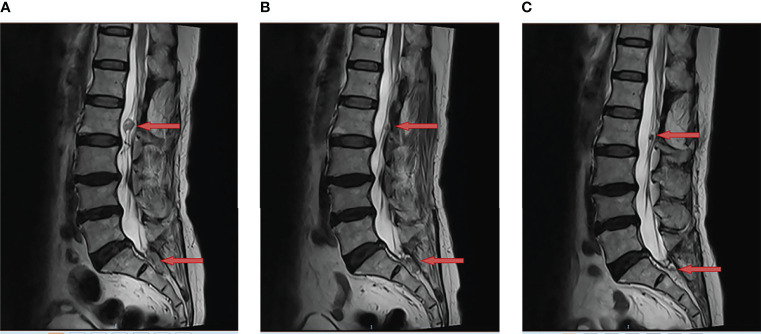
Anlotinib resulted in tumor regression of multiple recurrent lumbar and sacral cord hemangioblastoma. This is a sagittal magnetic resonance imaging (MRI) view of lumbar and sacral spine; **(A)** The two lesions at L2 and S1-S2 were observed to have increased in size (red arrow) in June 2021; **(B)** The MRI demonstrated tumor regression (red arrow) following one month of anlotinib in July 2021; **(C)** The MRI showed further regression in the enhancing tumor (red arrow) following three months of anlotinib in Sep 2021.

**Figure 3:**
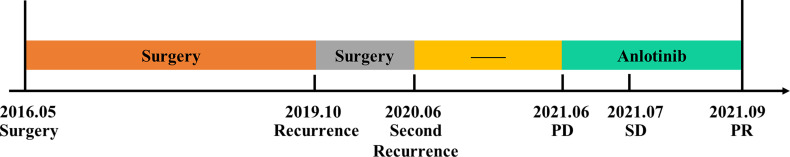
The treatment timeline of the patient with multiple recurrent hemangioblastoma.

In June 2021, tumor progression was observed. the MRI showed that the diameter of two lesions at L2 and S1-S2 increased to 1.8cm and 2.3cm respectively (**Figure 2A**). Despite the fact that the patient was symptom-free, she came to our department for further treatment. Anlotinib was begun after obtaining informed consent. Then, anlotinib (8mg qd d1-14, q3w) was orally administrated. There was a considerable improvement in the T2 signal change around the enhancing component, as well as a decrease in the size on the MRI from 1.8cm to 1.0cm at L2 and from 2.3cm to 2.0cm at S1-S2 after use of one month (**Figure 2B**). To date of Sep 22, 2021, the MRI demonstrated further decrease in the dimensions of residual lesions (**Figure 2C**). The patient was in good condition, evaluated as having partial response in accordance with the RECIST 1.1 system. In addition, apart from hypertension (grade I), no other adverse side effects of anlotinib such as hemorrhage, thrombopenia, fatigue and hypertriglyceridemia were observed in the patient. All of these adverse effects were evaluated pursuant to the Common Terminology Criteria for Adverse Events (CTCAE) Version 5.0. The treatment timeline of the patient is depicted in **Figure 3**. The patient has given her consent for the case report to be published.

## Discussion

HBs are benign and exceedingly vascular CNS tumors. They account for approximately 1.5% to 2.5% of all intracranial tumors, the majority of which are encountered in the cerebellar hemispheres (13). They occur both as sporadic disease and in association with VHL disease particularly in spinal manifestations (14). Sporadic HB in the absence of VHL disease is often curable with surgical resection, whereas there are still many patients suffering from recurrent or multifocal disease that is incompletely resected, normally treated with adjuvant RT (15). Despite their benign nature, repeated surgeries caused by a high recurrence rate are often of limited value and increase the clinical risk especially for patients with multiple recurrent lesions. There is no standard treatment beyond surgery for these types of HB particularly outside of CNS. Furthermore, the effectiveness of RT diminishes over time. Swelling after RT could also lead to neurologic decline and significant morbidity. Additionally, it has been reported that radiation necrosis, a complication following RT, can lead to poor outcomes for patients (6, 16). Therefore, developing new therapeutic options or drugs is of great significance for treatment of multiple recurrent HB.

The biological pathogenesis of HB is not completely elucidated; however, it has been reported that VHL disease is linked to a mutation of the VHL gene on chromosome 3p25-26 (17), leading to a stabilization of the hypoxia-inducible factor 1α protein and thereby to an up-regulation of target genes, which coding for VEGF, erythropoietin, platelet-derived growth factor, and so on (18). Considering that VEGF and its receptors seem to play a momentous part in HB tumor cell proliferation, we anticipated that using a high-selective and potent VEGFR inhibitor to block the VEGF signaling would alter the growth pattern of HB and consequently cause tumor regression. So far, progressive HBs successfully treated with VEGF-based anti-angiogenic therapy, such as monoclonal antibody bevacizumab, have been published (8, 19). In a single arm, phase II study with the VEGFR inhibitor sunitinib in patients with VHL disease, 9/11 patients with HB were evaluated as stable disease, whereas partial response was observed in 6/18 individuals with other lesions associated with VHL syndrome (20). Meanwhile, 1 open-label phase II study with sunitinib malate in patients who have VHL disease has reported partial response in 6/18 lesions (ClinicalTrials.gov Identifier: NCT00330564). Hence Anti-angiogenic therapy seems to be an attractive alternative for patients with multiple recurrent HB.

Anlotinib is a small molecular TKI inhibiting tumor angiogenesis and proliferation signaling. In clinical applications, it has exhibited satisfactory efficacy against diverse solid tumors, containing NSCLC, breast cancer, soft-tissue sarcoma, renal cell cancer and so on. Anlotinib has been approved by the Chinese Food and Drug Administration for third-line treatment for advanced NSCLC patients. Also, anlotinib has exhibited inspiring efficacy as well as low toxicity in heavily pre-treated HER2-negative metastatic breast cancer (21).

In this case report, we first delineate the significant effectiveness of anlotinib in the patient with multiple recurrent HB. Moreover, anlotinib showed few adverse effects, ensuring their life quality while increasing the treatment efficacy. The clinical benefit is hard to explain but it could be attributed to a reduction in perfusion and edema, as well as improved perfusion of adjacent brain parenchyma. Possible mechanisms are the known decreases in capillary density, microvascular flow, and blood vessel permeability in response to anlotinib. We believe that anlotinib merits further investigation in order to better define its role in patients with this challenging disease. In addition, more clinical trials are still needed to determine whether the effect of anlotinib on multiple recurrent HB will translate into improvements in disease control, including prolonged progression-free survival (PFS) and overall survival (OS).

## Conclusion

No reports were found in a Medline search (keywords: anlotinib; hemangioblastoma) that showed HB may be in response to anlotinib favorably. As far as we are aware, our case is the first report that demonstrates anlotinib induces a positive radiographic response when used to treat multiple recurrent lumbar and sacral cord HBs. The mechanism underlying this remains scanty. Additionally, the effect of anlotinib in treating multiple recurrent HB is yet to be determined, including whether it will improve PFS and OS, but we believe this case report will provide indication for future research.

## Data Availability Statement

The original contributions presented in the study are included in the article/supplementary material. Further inquiries can be directed to the corresponding authors.

## Ethics Statement

Ethical review and approval was not required for the study on human participants in accordance with the local legislation and institutional requirements. The patients/participants provided their written informed consent to participate in this study. Written informed consent was obtained from the individual(s) for the publication of any potentially identifiable images or data included in this article.

## Author Contributions

NJ and CS performed the data acquisition and manuscript drafting. YH and XW completed the radiological images analysis. YY and WL reviewed the manuscript. All authors contributed to the article and approved the submitted version.

## Funding

This case report was financially supported by the National Key Research and Development Program of China (ZDZX2017ZL-01), High-level Innovation Team of Nanjing Medical University (JX102GSP201727), Wu Jieping Foundation (320.6750.17006), Key Medical Talents (ZDRCA2016023), 333 Project of Jiangsu Province (BRA2017534 and BRA2015470), The Collaborative Innovation Center for Tumor Individualization Focuses on Open Topics (JX21817902/008) and Project of China Key Research and Development Program Precision Medicine Research (2016YFC0905901).

## Conflict of Interest

The authors declare that the research was conducted in the absence of any commercial or financial relationships that could be construed as a potential conflict of interest.

## Publisher’s Note

All claims expressed in this article are solely those of the authors and do not necessarily represent those of their affiliated organizations, or those of the publisher, the editors and the reviewers. Any product that may be evaluated in this article, or claim that may be made by its manufacturer, is not guaranteed or endorsed by the publisher.
